# An interview with Mario Polo

**DOI:** 10.1590/2177-6709.22.6.014-024.int

**Published:** 2017

**Authors:** Mario Polo

**Affiliations:** » Received his orthodontic training at the University of Texas in Houston, Texas Medical Center. » Is board-certified in orthodontics by the American Board of Orthodontics. » Has been inducted as a Fellow of the International College of Dentists. » Is an Associate Professor in the School of Dental Medicine at the University of Puerto Rico. » Has contributed as a Guest Lecturer at the University of Texas, the University of Maryland, the University of Puerto Rico, and the Virginia Commonwealth University. » Has devoted over 40 years as an orthodontic educator, researcher, and clinician. » Has served as a Reviewer in the AJO-DO, the Angle Orthodontist, the Aesthetic Surgery Journal, and the Journal of Plastic, Reconstructive, and Aesthetic Surgery, among others. » Has served the American Association of Orthodontics in multiple elected and appointed positions. » Is the pioneer in the use of Botox for the correction of excessive gingival display. » Has performed more than 60 Onabotulinumtoxin A-related international presentations and publications, having his major publications received over 300 reference cites. » Was awarded the prestigious Gerald A. Devlin Award by the Middle Atlantic Society of Orthodontists Constituency of the American Association of Orthodontists.

**Figure:**
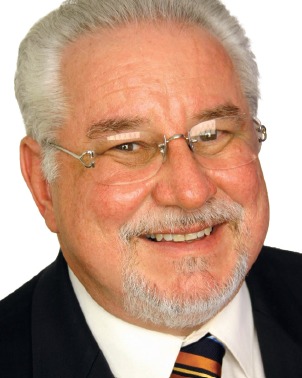

I got acquainted to Dr. Mario Polo a couple of years ago through Dr. Mucha, who forwarded me an e-mail of Dr. Polo in which he demonstrated interest in sharing his knowledge on Botox treatment for gummy smiles in São Paulo, on a trip where he was accompanying his son. I thought that was an invaluable opportunity and arranged together with the Brazilian Association of Orthodontics in São Paulo an adequate lecture opportunity for him, which unfortunately I could not attend to, but was extremely appreciated by the attendees. We did finally meet personally in San Francisco during an AAO Meeting, where I could certify myself of Dr. Polo’s enthusiasm for his profession and great contribution for the specialty of Orthodontics. Dr. Mario Polo was born in a small suburb of San Juan, Puerto Rico. After attending the University of Puerto Rico School of Dental Medicine in San Juan, he continued his studies at the University of Texas School of Dentistry, where he received a Master of Science degree and specialty certificates in Pediatric Dentistry and in Orthodontics. Now in practice for 40 years, 20 of which he has been providing orthodontic and cosmetic treatment to participants of several international beauty pageants, he also is an Assistant Professor at the Postgraduate Department of Orthodontics of the University of Puerto Rico. Esthetics has always been his passion, both surgical and non-surgical / orthodontic. He has presided several local and national dental and orthodontic associations, and in 2011 was awarded the prestigious Gerald A. Devlin Award by the Middle Atlantic constituency of the American Association of Orthodontists. Sometimes referred to as “The Father of Botox in Dentistry”, his life made a big change in 2001 with his idea and eventual research about using Botox for smile improvement, something that gave rise to multiple international publications and presentations. Recently, he published a textbook chapter in Sergio Kahn’s textbook *“Sorriso Gengival”* and is currently working in the editing on a textbook on Botox as related to smile esthetics. He is a reviewer in several international orthodontic and plastic surgery journals. Dr. Polo has been married for 45 years, is the father of two sons (a neurointerventional surgeon in Houston, TX and a digital-media producer with National Geographic in Washington, DC), grandfather of three boys, and anxiously waiting for a granddaughter. In his free time, Dr. Polo enjoys nautical navigation, deep-sea fishing, and traveling. I hope you enjoy reading this interview and can appreciate Botox as an alternative treatment for excessive gingival exposure in smiling.

Conheci o Dr. Mario Polo há alguns anos, por intermédio do Dr. Nelson Mucha, que me encaminhou um e-mail do Dr. Polo, no qual ele demonstrava interesse em compartilhar seu conhecimento sobre o tratamento com Botox^®^ para sorrisos gengivais, em São Paulo, em uma viagem na qual que acompanhava seu filho. Acreditei ser uma oportunidade única e organizei, junto com a Associação Brasileira de Ortodontia, uma palestra adequada a ele - à qual, infelizmente, não pude comparecer, mas que foi extremamente apreciada pelos participantes. Nós finalmente nos conhecemos pessoalmente em São Francisco, durante uma reunião da AAO, onde pude certificar-me do entusiasmo do Dr. Polo por sua profissão e sua excelente contribuição para a especialidade da Ortodontia. Dr. Mario Polo nasceu em um pequeno subúrbio de San Juan, Porto Rico. Depois de frequentar a Faculdade de Odontologia de San Juan, da Universidade de Porto Rico, continuou seus estudos na Faculdade de Odontologia da Universidade do Texas, onde obteve os diplomas de Especialista e Mestre em Odontopediatria e Ortodontia. Agora, na prática há 40 anos, dos quais 20 oferecendo tratamento ortodôntico e cosmético a participantes de vários concursos internacionais de beleza, ele também é professor assistente no Departamento de Pós-graduação em Ortodontia da Universidade de Porto Rico. A estética sempre foi sua paixão, tanto cirúrgica quanto não cirúrgica e ortodôntica. Ele presidiu várias associações de Odontologia e Ortodontia, locais e nacionais e, em 2011, recebeu o prestigiado prêmio Gerald A. Devlin, concedido pelo eleitorado do Atlântico Médio da Associação Americana de Ortodontistas. Por vezes citado como “O Pai do Botox na Odontologia”, sua vida teve uma grande mudança em 2001, com sua ideia e eventual pesquisa sobre o uso do Botox^®^ para melhorar o sorriso, algo que deu origem a múltiplas publicações e apresentações internacionais. Recentemente, publicou um capítulo no livro ‘*Sorriso Gengival*’, de Sergio Kahn, e está, atualmente, trabalhando na edição de um livro sobre Botox^®^ relacionado à estética do sorriso. Ele é revisor de vários periódicos internacionais de Cirurgia, Ortodontia e Plástica. O Dr. Polo é casado há 45 anos, é pai de dois filhos (um neurocirurgião em Houston/TX, e um produtor de mídias digitais para a *National Geographic*, em Washington/DC), avô de três meninos e ansiosamente à espera de uma neta. Em seu tempo livre, o Dr. Polo aprecia navegação náutica, pesca em alto mar e viagens. Espero que gostem de ler esta entrevista e possam apreciar o Botox^®^ como um tratamento alternativo para exposição gengival excessiva ao sorrir.

Flavia Artese - interview coordinator / coordenadora da entrevista

**How and when did you have the idea of using Botulinum toxin type A (BTX-A) for reducing the hypermobility of the upper lip during smiling? (Flavia Artese)**

**Figure 1:**
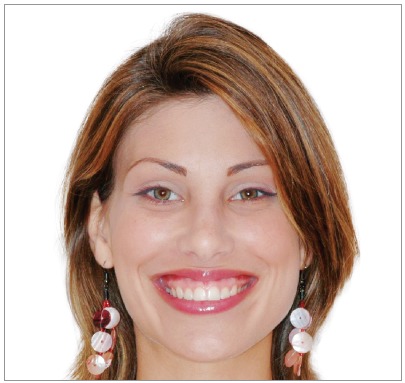
Smiling photo of the patient who motivated Dr. Polo to seek a treatment alternative for the correction of gummy smiles using Botox^®^.

**Figure 2:**
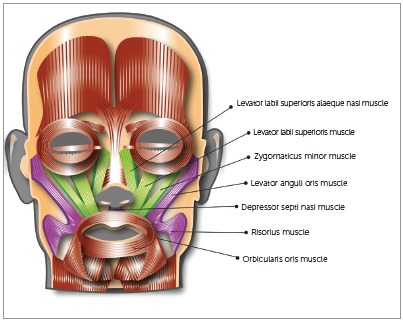
Diagram of facial muscles. Those injected with Botox following Dr. Polo’s protocol (LLSAN, LLS, and Zmi) are highlighted in green.

Back in 2001, I was invited to a fashion show where the competition trousseau for that year’s Miss Puerto Rico Universe, a patient of mine, was to be presented to the press and the public. One of the models, another patient of mine, was part of the group modeling the outfits, and upon finishing the show, she smiled and I noticed that even though I had performed an orthodontic treatment with excellent dental results, there was still something about her smile that I didn’t liked. My patient was having an excessive gingival display (EGD) when smiling. The fact that she had a short upper lip just immediately hit my mind (Fig 1). I went back to my office the following day, re-evaluated her photos and radiographs, ruled-out other etiologic factors in her condition, and concluded that her “gummy smile” was due to excessive muscle pull of her upper lip by the lip’s elevator muscles, mainly the *Levator labii superioris alaeque nasi* (LLSAN), the *Levator labii superioris* (LLS), and the *Zygomaticus minor* (Zmi) (Fig 2). 

At that time, Botox (BTX-A) was being used to correct facial rhytides. Researching on the subject, I found that this correction was accomplished by chemodenervation attained by Botox on the muscles producing the frown lines or crow’s feet in the glabellar and periocular areas of these patients. In other words, Botox was partially paralyzing muscles in those two other anatomical regions of the face, and smoothing out the creases created by their contraction. 

2 + 2 = 4 ! If it was used for the other purpose, BTX-A could also be used for the purpose I had in mind: to diminish excessive contraction of other muscles in my anatomical region of interest: the upper lip elevator muscles.

My hypothesis was developed, initial research took place on a pilot study with five subjects, one of them being my muse, serendipity happened, and the rest is history.

By the way, I prefer the term “hyper-contractibility” (or “hyper-functional” ) than the term hypermobility: it makes better reference to the etiology of this condition, a neuromuscular situation where excessive muscle contraction takes place. For me, the term hypermobility is more allusive to a description of lips being able to be moved in all directions: up, down, sideways, i.e., like in flabby or flaccid lips. In these cases with excessive gingival display, I visualize the upper lip moving only in one direction: upwards. In other words, I find the term “hyper-contractibility” better descriptive.

**Has your injection protocol remained the same as the one reported in 2005, and again in 2008? (Flavia Artese)**

**Figure 3:**
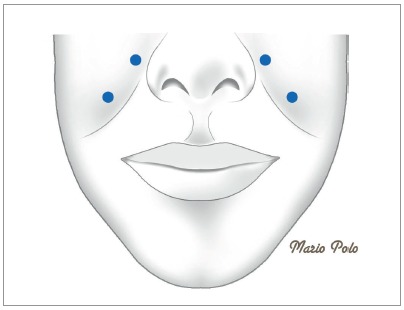
Diagram depicting approximate location of the Polo Injection Points, corresponding to the LLSAN/LLS and the LLS/Zmi sites. Final location of the injection sites are determined by muscle contraction palpation and by muscle functional animation (smile production).

**Figure 4:**
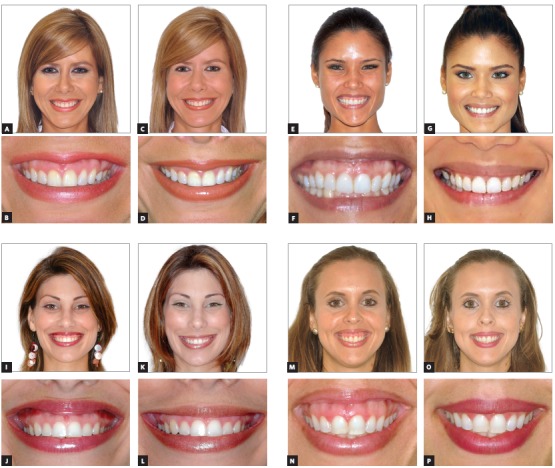
Before and after photos of patients with four levels of gingival display injected with Dr. Polo’s current injection protocol, as described in Table 1. A-D) Level 1; E-H) Level 2; I-L) Level 3 and M-P) Level 4.

My injection protocol has been modified from the one initially reported in the AJO-DO in 2005^1^ and eventually used for the research leading to the article published in 2008.^2^ For the five patients injected during Phase III of the research that gave rise to the 2005 publication, I gradually increased the dose from 1.25 U during Phase 1 to 2.5 U during Phase 3 at the *Levator labii superioris alaeque nasi* (LLSAN) /*Levator labii superioris* (LLS), and the *Levator labii superioris*(LLS) /*Zygomaticus minor* (Zmi) sites on both sides of the face^1^ (Fig 3). This same dose and sites were selected to be the ones used on the research that was conducted in 2005-2007 and published later, in 2008.^2^ Being a controlled study, all 30 patients in the sample had to receive equal doses at exactly the same sites, regardless of the amount of gingival display present. That’s the reason why 21 subjects were at the 0-2mm level post injection, and in 9 others, the target was overshoot, thus resulting in an upper lip position below the dento-gingival junction. After publication of the results attained, I went through a dose modification process, which, after being reviewed twice, the current injection protocol was established and published.^3^ ^,^ ^4^ Based on the amount of exposed gingival tissue when smiling, four doses are currently used with much esthetic success being accomplished (Table 1). Please refer to before and after photos (Fig 4) of four individuals with GE levels corresponding to, and treated with these four doses.

**Table 1:** Current injection protocol, according to the amount of gingival exposure.

Gingival exposure	Number of injection sites and its location	Dose (by side)	Total Units
4.0 - 5.0 mm	1 (LLSAN/LLS)	2.0 U / location	4.0 U
5.0 - 7.0 mm	1 (LLSAN/LLS)	2.5 U / location	5.0 U
7.0 - 8.5 mm	2 (LLSAN/LLS; LLS/ZMi)	2.0 U / location	8.0 U
> 8.5 mm	2 (LLSAN/LLS; LLS/ZMi)	2.5 U / location	10.0 U

**What would be your objective criteria to indicate surgery or BTX-A injection in patients with gummy smiles? (Nelson Mucha)**

There are different types of procedures that could be considered as surgical treatment options for the management of excessive gingival display (EGD). If skeletal in nature, like the gummy smiles observed in Vertical Maxillary Excess (VME), the Le Fort I osteotomy with impaction is the treatment of choice. However, as later explained, BTX-A injections for VME remains as an alternate treatment. 

For those with a gingival-related etiology, if due to altered passive eruption (APE), there is the choice of several modalities of esthetic surgical crown lengthening (SCL) procedures: either gingivectomy, gingivectomy with osseous reduction, apically positioned flap surgery, or apically positioned flap together with osseous reduction surgery. The choice of these alternatives depends on the amount of keratinized gingival tissue and the location of the mucogingival junction relative to the alveolar bone crest. Post-operative gingival growth recurrence is a factor to be assessed, since lack of long-term stability in some cases has been observed and reported in the periodontal literature (mostly in the gingivectomies-only group).

Regarding SCL procedures, I do use them in cases where the etiological factor responsible for the EGD is Altered Passive Eruption, with obvious short clinical dental crowns, and where the tissue removal will not cause additional esthetic problems, with the creation of dark triangles or extremely long, apically tapered clinical crowns. EGD’s in the range of 3.0-5.0 mm meeting the above-stated criteria are referred for SCL and BTX-A is not injected. There are some cases with combined etiology, altered passive eruption and muscle hypercontractibility, where I treat both by initially injecting BTX-A, followed with SCL. The selected BTX-A dose should contemplate further dental display reduction with the SCL procedure, and should be planned ahead with the periodontist to perform the surgical part of this combination procedure. Highly aesthetic results are attained. Such a Case Report is in progress for publication.

Mucosal elliptical excision was first introduced by Rubinstein and Kostaniovsky^5^ in 1973. Litton and Fournier^6^ followed in 1979 with the lip repositioning surgery technique, and then Miskinyar^7^ in 1984 reported cases treated with myectomies. Stability became an issue, and was addressed by Ellenbogen and Swara^8^ in 1984. Lip Repositioning Surgery (LRS) for “hypermobility”, basically is a mucosal elliptical excision procedure, as first reported back in 1973, almost 50 years ago. We must first address this question from the viewpoint of the existing controversy regarding stability of the procedure, regardless various techniques (technique modifications) of the procedure now being employed by several clinicians or authors. Although some claim good stability, most of these articles appearing on the literature are retrospective case reports with a very limited sample, many with merely one subject, and with a short period of time to adequately assess long-term stability.

Peres et al,^9^ a São Paulo group of periodontists, recently published results of a systematic evaluation of published articles on LRS which best addresses this conflicting issue. The purpose of their study was to evaluate if LRS improved the long-term smile outcome and dental esthetics. Nine articles were evaluated: Their *n* values were 1, 1, 14, 1, 1, 1, 2, 7, 13. Cases were re-evaluated for stability at 6 months in six of these reports, in 12 months in two of them, and 1 article’s report (*n*= 7) ranged from 6 months to 3 years - only 1 subject of this particular sample was followed up at a T_2_ of 3 years. Their conclusion: available data was based mostly on clinical case reports; it had a reduced number of subjects; it was based on low level of scientific evidence; it did not have enough scientific evidence regarding predictability, and there is a need for additional studies to further evaluate esthetic results of LRS regarding predictability and long-term results.

**Figure 5:**
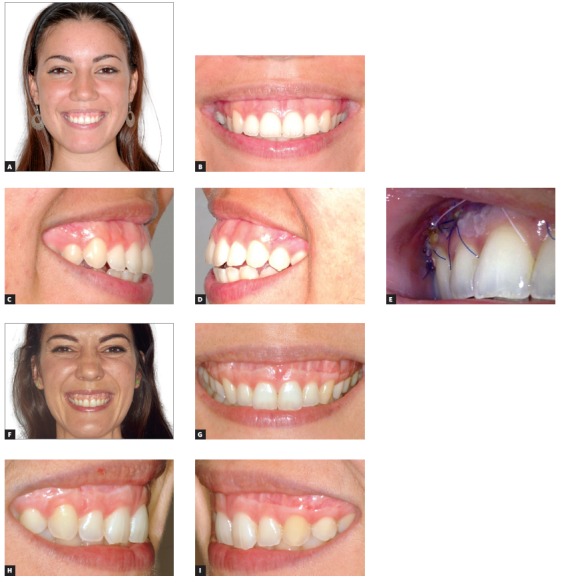
Serial photos of a patient with gummy smile treated with Lip Repositioning Surgery (LRS). Relapsed 15 months after procedure.

My personal experience with LRS has been negative because of issues related to complications, stability, and esthetic results attained. Figure 5 shows a patient with unstable results whose relapse produced a permanent scar now quite visible when smiling. She initially had one problem, and now, she (and other patients as well) has two different problems. At 3 years post LRS, the scar is still present and is visible when smiling. 

(photos supplied by the patient). A) Before LRS, May, 2014. B) After LRS while sutures still in place, May 2014. C) 12 months post-LRS procedure, August, 2015.

I objectively discuss this treatment alternative with my patients, present pros and cons, document my position regarding its use by means of A-V material, and let the patient select how they want to proceed. Personally, I don’t support its use because it is an invasive camouflage procedure with questionable long-term stability. LRS does not target the root of the etiology of this condition. Hence, LRS is likely doomed to fail, as the condition tends to recur in a significant number of individuals.

A research project aimed at providing a permanent result with adequate stability for the correction of EGD with a neuromuscular etiology is now being performed at the University of Puerto Rico Medical Science Campus under my mentorship. We are directing correction at the root of the condition: the hyper contractibility of the LLSAN, the LLS, and the Zmi muscles.

There are times where a combination of procedures might be indicated. Altered Passive Eruption and excess muscular contraction could both present simultaneously. On these cases, I recommend to first undergo Botox injections, and perform SCL two weeks afterwards. Results with these combination technique produces results. 

**Figure 6:**
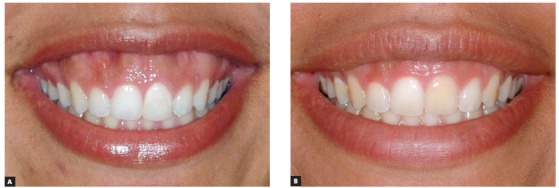
Before and after photos of a patient with a severe gummy smile and skeletal vertical discrepancy. Surgical correction rejected: treated with BTX-A Dose #4.

There are also times where BTX-A could offer an alternate temporary solution for VME cases. As you will observe in photos of individuals I have treated with Botox, it can correct extreme levels of excessive gingival display, levels up to parameters observed in VME cases (Fig 6). For these type of cases, if the patient rejects a LeFort I osteotomy, I do not hesitate to offer them the use of Botox for the temporary correction of excess gingival display when smiling. It is widely accepted.

**At what age can BTX-A start to be used? (Mayra Seixas)**

Qualified professionals will not treat anyone under the age of 18, unless Botox is used to treat specific medical conditions. Onabotulinumtoxin A is used by some pediatric specialists to treat upper and lower limb spasticity and other medical conditions during childhood. For the correction of excessive gingival display, I use it on individuals past 18 years of age, and I require parental or tutorial consent in order to perform treatment.

**Within the 40 to 60 year-old age group, how do you vary your injection protocol for the correction of EGD, as determined by dose and the Polo Injection Sites? (Victor Acevedo)**

Age is not a criterion used to determine injection dose. Dose is solely determined by the gingival’s display degree of severity. You may have a 40-60 year old individual requiring the same dose for GD correction as a 25-30 year old subject. However, we know that as we age we tend to show less our dentition when smiling. This sagging, or ptosis of the upper lip, is due to losing skin laxity and muscle tone as part of the aging process. Thus, you might get a 40-60 year old individual in which you might have to use a lower dose than what you could be using with a younger individual: you choose your dose based on the amount of gingiva exposed, dictated by the amount of contraction and elevation of the upper lip musculature, NOT by the age of the patient. 

I do have noticed that in patients which I have been injecting on a continuous basis for over 10 years, now within this age range of 40-60 years, the dose I now use with them is less than the one used with them at a younger age. Personally, I feel that this dose reduction is due to the reason previously explained and is not due to a localized effect of the toxin at the site. I base this conclusion on clinical observations of similar effects in other facial anatomical areas, areas not injected with the toxin, on a similar frequent basis.

**Based on your cumulative sample of patients of over 750 individuals treated with BTX-A for the correction of excessive gingival display (EGD) due to hypercontractibility of upper lip elevator muscles on a long-term and repetitive basis, what is the largest cohort of patients having received BTX-A repeatedly for a long-term for this treatment modality? (Victor Acevedo)**

The number of individuals has now increased from the amount you refer to and reported in 2016.^4^ Several cohorts have resulted along the 15 years that I have been injecting BTX-A for the temporary correction of gummy smiles. The one with the longest number of injection sessions contains 1 individual who has had 22 injection sessions within 11 years she has been under my care. Most individuals don’t mind being injected twice a year, as long as they can correct their smiles - at times, embarrassing. This individual started injecting at the age of 32 and received the maximum dose I use in my protocol previously described: 2.5 U at 4 sites. Now, at age 43, she only received the minimum dose of 2.0 U at only 2 sites, and is soon to need none. Over the span of these 11 years, she has paid an average of US $150.00 per session, for a total of US $3,300.00, which compares very good for the US $3,000.00 average fee charged in my community to perform a LRS procedure.

**According to one of your papers,** ^**2**^ **the BTX-A effects in patients with gummy smiles would last approximately 30-32 weeks. How do you communicate this to the patient and how do they generally react to the need for reapplication? (Nelson Mucha)**

Since Puerto Rico is a territory of the United States of America, and according to federal law of the USA, I need to tell my patients that Botox has been approved for cosmetic applications in the glabellar and periocular regions, that I’m using it as an Off-Label Indication for the temporary correction of their condition, and (as exactly as written in Allergan’s Inc. [Irvine, CA, USA] Onabotulinumtoxin A [Botox, ONA] vial package’s insert), that it’s temporary action lasts for up to 4 months.

Its temporary duration for the treatment of glabellar and periocular rhytides varies from individual to individual, and some patients report its effect for these two purposes lasting only for 2 months, others up to 6 months.

In my research reported in 2008,^2^ its effect lasted for up to 6 months in most individuals, in some less than 6, and in some, the baseline GE level had not been reached back when evaluating all subjects at the end of the experimental 6-month period. Based on these facts, a third-order polynomial equation graph was constructed; the curve there described suggests that possibly results could last up to 30-32 weeks in a very small percentage of the population.

Nonetheless, patients are well-informed, verbally and in written, and they do know that results are temporary and variable. A huge majority don’t mind this temporary effect and accept the injection procedure, while maybe 1 or 2 individuals might reject it.

**Have you clinically noticed muscle mass reduction due to disuse of targeted muscles in subjects you have injected for EGD correction? (Victor Acevedo)**

I have not clinically noticed any muscle mass reduction in naso-zygomatic-labial region where injections are done for EGD correction, nor have I had any patient complaining of any visible change in this area. Muscle mass reduction has been reported and is frequently used for facial reshaping in the cosmetic treatment of patients with masseter hypertrophy. Contrary to treatment protocols for treatment of crow’s feet and mostly for my treatment protocol for EGD, where relatively low doses are used, the protocols for masseter hypertrophy correction call for high doses performed on these thick muscles over a several-year span so as to maintain part of the total mass reduction attained. 

Kim, Park and Park^10^ published an excellent article on this subject where masseter mass reduction was clinically observed and measured. A mean of 240 U of Abobotulinumtoxin A (Ipsen Biopharm Ltd., Werexham, UK), another commercially available product similar to Botox (Onabotulinumtoxin A, Allergan, CA, USA) were injected in both masseter muscles of 121 individuals over a 4.28 year-period, 5-8 sessions every 6 months, and muscle mass thickness was measured with ultrasound technology. Since there is a dose variation between these two botulinum-A toxins, it is worth stating that 240 Abo Units are equivalent to roughly 100 Ona Units. Over this time frame, approximately 1000 Abo Units were injected, equivalent to ± 400 Ona Units. From baseline to T_2_, the mean masseter thickness was reduced approximately 2.2 mm, and continued from T_2_ to T_8_ to reduce in thickness an additional 2.8 mm (mean), for a total of approximately 5.0 mm (T_0_-T_8_).

The patient to which I refer on a previous question, the one having received the greatest BTX-A, has only received 168 Ona U, at 3 different target muscles, during 22 injection sessions, over an 11-year period, a very small amount indeed. An evaluation to measure muscle mass decrease in the LLSAN, LLS, and Zmi area has also been undertaken. 

**The reapplication of BTX-A injection would have undesirable effects or contraindications and how many reapplication would be possible? (Nelson Mucha)**

There are several contraindications to use BTX-A: past history of hypersensitivity to Botox or other commercially available neurotoxins, hypersensitivity to any of the components in its preparation, including allergy to eggs (albumin-related), infection at the injection site, concomitant debilitating neuromuscular diseases, presence of upper respiratory infections, pregnant women (may cause fetal harm), and is to be used with caution in presence of compromised respiratory function. Please refer to the vial’s package insert for additional information prior to use. 

BTX-A reapplications should be performed after the toxin has been completely metabolized and no longer present in the system, usually four months, as previously addressed. The main complication arising from injections performed within less than this time lapse is the production of antibodies which might eventually have the potential to lead to botulinum neurotoxins (BNT’s) non-responsiveness. The application of “touch-ups” or “boosters”within a short treatment interval of 2-3 weeks, or the re-injection requested by some cosmetic patients to be performed “before loosing the attained effect” too close to prior injection session, could most likely produce secondary immunogenicity.^11^ We need to keep away from performing touch-ups, boosters, or short interval interjection sessions.

Primary non-responsiveness to BNT’s has been reported and has happened once in my practice with gummy smile patients. Factors affecting immunogenicity could be product- or treatment-related, all having to do with multiple immune response factors.^11^

Regarding side-effects related to the frequency in its use, up to what I recall, Allergan has not reported any in their package inserts, nor in their “Important Safety Information” appearing on their websites, nor have I been able to find any related publication in PubMed. Please bear in mind that BTX-A has been in use for medical therapeutic treatment of blepharospasm and strabismus, under USA’s FDA approval, since 1989. However, potential injectors are reminded it is their own responsibility to review all current literature and updates regarding side-effects and complications prior to injecting the toxin.

As far as how many reapplications, because of the past global experience with BNT’s relating them to negative side-effects from multiple exposure to the toxin, despite its history of prolonged use and larger doses used for other cosmetic and therapeutic indications, I feel comfortable using it as per my protocol on what I call as being “the normal course of the gummy smile condition”, since once detected, until it resolves as part of the normal aging process, or until a stable surgical correction technique is found. Based on my experience, this period could last from age 18 until age 45. Many individuals receive BTX-A for facial cosmetic purposes for an equal amount of time: on the average, from age 40 until age 65, at which time, they have to resort to the scalpel! 

**Do you adjust your injection protocol based on ethnicity? (Victor Acevedo)**

No. Ethnicity is not the variable to treat: the variable is the amount of gingival exposure secondary to the amount of muscular contraction of the upper lip elevator muscles.

In Puerto Rico we have four ethnic groups (White, Black, Indian, and Mestizos or Mulatos, while in Brazil there are five main groups (White, Black, Indian, Asian, and Pardos, and within the Pardos you find five additional sub-ethnic groups: Mulatto, Cafuso, Caboclo, Juçara, and Ainocô).

I personally find a racial, not ethnic, predisposition to gummy smiles, short upper lip, and upper lip “hypermobility”, with Hispanics and Asians showing the highest prevalence, followed by Whites, and Blacks presenting the smallest GS incidence. There are no studies to back-up this observation. As is the case with ethnic groups, neither do I adjust doses based on races. 

**What experience do you have with the use of BTX-A to reduce hypermobility of the lower lip in cases with excessive lower incisor display during smiling? (Mayra Seixas)**

I have used BTX-A to treat excessive lower incisor display and asymmetric smiles caused by hyper contractibility mostly of the *Depressor anguli oris*(DAO) and less often, the *Depressor labii inferioris*(DLI). My protocol is similar to the first two doses employed for upper lip contraction control, since problems of this nature on the lower lip are smaller in intensity than on the upper. Dose ranges from 2.5 to 5.0 U, depending on amount of inferior lip retraction. 

The incidence of gummy smile in the lower anterior portion is almost non-existent, and when present, it may be related to a lack of muscle tone or hypotonicity, something to watch for before injecting a strong chemodenervator like BTX-A, which might even aggravate the condition.

**What advice would you give to an orthodontist that would like to start using BTX-A in their clinical practice? (Mayra Seixas)**

Read, read, and then read more. Get trained!

A thorough review of the anatomy is of utmost importance. You must look at a face and practically see what is laying behind the skin. Muscles, arteries, veins, fatty compartments, glands, bone… muscle origins and insertions, how they run across the face, their muscle mass, length and thickness, proximity to vital structures… You really have to know your anatomy quite good, or you are doomed to fail.

Besides anatomy, in order to familiarize yourself with neurotoxins, read anything you can find in the literature related to BTX-A’s mode of action, physiology, myophysiology, pharmacology, immunology, and pathology.

But reading is not enough. You have to adequately train yourself to do it. Get all the legal permits, licenses, and malpractice insurance coverages to cover yourself if something goes wrong.

Subscribe to surgical and dermatologic journals, and continue reading.

Once ready and doing it, just like you do in orthodontics, go to Continuing Education courses and meetings to keep abreast of the latest findings in this field.

**In Brazil there are regulatory issues and debates between the medical and dental associations regarding the application of BTX-A by dentists. The current situation allows dentists to use BTX-A and filling substances limited to the anatomical area of professional reach. What are your opinions regarding this subject? (Flavia Artese)**

The application of BNT’s and fillers is government-regulated worldwide and conflicting issues are so present. I personally feel that this might most likely be due by lack of knowledge within the medical community on the competency of a 21^st^ Century dental surgeon in medical matters. In having conversations with an immense number of physicians, it has become apparent that they didn’t realize we, as dentists were so well-prepared in so many aspects of medicine. As modern dentists, we have the basic training needed to eventually master the art and science of the application of neuroregulators and dermal fillers after eventually attending special courses designed to train healthcare professionals. Our anatomical area is the head and neck region. We are one of the specialties that knows best the anatomy of this region. As general dentists, we are trained to inject anesthetic solutions within the area. We are an esthetically-oriented sub-group of healthcare providers, without loosing the perspective of maintaining optimal health and gnathological function and balance within the oral cavity and adjacent anatomical structures. The attributes go on and on and on. Besides, as specialists within dentistry, we as orthodontists, as periodontists, and as oral/maxillofacial surgeons, have even gone further in augmenting our knowledge in medical basic sciences, such as head and neck anatomy.

However, it is up to us, as dentists, to let the medical community and appropriate legislative bodies know who we are and what we can do. If we don’t do it, no one else will. 

When I first started incurring in this field, only three states within the United States of America allowed dentists to inject BNT’s and fillers. The exposure we gained within the medical community with our research, publications and presentations, spoke highly on what we “as dentists”, had to offer within this treatment modality. Physicians slowly started changing their minds on the way we were looked upon by them. Communities started changing the way they looked upon us. Within the USA, close to 40 states now allow adequately trained dentists to perform these procedures. The world started looking at us with a new perspective. 

Education is the mother of knowledge. With determination, we can do it.

It only takes one person, and a lot of courage.
